# Transdisciplinarity as an Inference Technique to Achieve a Better Understanding in the Health and Environmental Sciences

**DOI:** 10.3390/ijerph7062692

**Published:** 2010-06-21

**Authors:** Matilda Annerstedt

**Affiliations:** Area of Work Science, Business Economics and Environmental Psychology, Department of Landscape Planning, Swedish University of Agricultural Sciences, Box 88, 230 53 Alnarp, Sweden; E-Mail: matilda.annerstedt@ltj.slu.se; Tel.: +46-4041-5078; Fax: +46-4041-5010

**Keywords:** integrative patterns, cooperation, ecology, human behaviour, philosophy of science, collaboration, sustainability, methodology, globalisation

## Abstract

The problems of the world are not categorised into disciplines. They are far more complex, a reality that the tradition of transdisciplinary research has recognised. When faced with questions in public health and sustainability, the traditional scientific paradigm often seems inadequate, and, at least in medicine, transdisciplinary research has not yet been fully appreciated or acknowledged. This lack of recognition may be partly caused by a lack of cooperation between disciplines and between science and society. In this paper, I discuss some of the challenges that scientists and policymakers face in public health and environment within a methodological context. I present transdisciplinarity as a modern research tool that should be applied in research in health and the environment and argue that these topics can be approached beyond the inherent obstacle of incommensurability between disciplines. Thus, a small step might be taken in this immense research arena.

## 1. Introduction

In an increasingly globalised world, the search for knowledge faces new challenges. Public health and preserving the environment are global issues that raise endless questions and dilemmas for both researchers and society. Through a genuine collaboration between these two disciplines, which, on the surface, seem rather disparate, we may find new answers and achieve a better understanding of how the interactions between human beings and their environment shape or distort our society. This understanding could be informed by policy and used by decision makers when they deal with issues of health and the environment in a modern and sustainable manner.

To understand the complexity of the concept of health in all its dimensions, one requires not only knowledge about human biology, but also to at least the same extent, knowledge about society and the environment. Many contemporary health issues are disorders that are affected by social and environmental factors and can result in mental or behavioural disorders [1]. The search for solutions requires collaboration across any constructed barrier between diverse instances. When we reflect on several innovative approaches to problems in which solutions to health problems also become partial solutions to environmental problems, the innate bridge between health and human behaviour becomes clear. This link is expressed by human biopsychology (e.g., neurological networks, hormonal mechanisms and psychological functions) and by our surroundings (e.g., ecological environment and social concepts).

The issues of natural resource management and environmental degradation are at least as dynamic and multifaceted; they require updated scientific knowledge from diverse disciplines that range from plant genetics to the economics of forest management. Shrader-Frechett and McCoy [2] dealt with one aspect of this complexity and highlighted the absence of uniform empirical laws across species or communities.

Researchers have expressed attempts to embrace a more complex knowledge in various terms. The concept of interdisciplinary research was first mentioned in the 1940s [3] in the fields of psychology and epidemiology. Interdisciplinary and multidisciplinary research have since come to involve separate input from different disciplines, but without creative attempts to blend these approaches for a more profound understanding of the problem or its potential solutions [4]. The term multidisciplinarity refers to several disciplines. Some scholars have questioned, over the years, the simple application of other disciplines, such as psychology and sociology, to public health and epidemiological research. It appears that this approach is not practical without a serious effort to bring people together to work collaboratively [5]. In interdisciplinary research, there should be a stronger focus on the integration of methodological and/or theoretical components between disciplines [6]. However, collaboration is still restricted to dealing with limited knowledge bases and predetermined problems.

The term transdisciplinary research (TR) first emerged in the 1970s [7], and today there are several definitions for the term. These definitions share the idea that in TR, compared to inductive or deductive paradigms, the *problem context* and *knowledge production* are closely linked and it includes a team approach to science that aims for synergy from the phases of problem defining to solutions. It should also involve the integration of theoretical and methodological perspectives from different disciplines to develop novel conceptual and empirical analyses of a research problem [4,8]. For complex health issues, it is hoped that TR can provide “a systematic, comprehensive, theoretical framework for the definition and analysis of the social, economic, political, environmental, and institutional factors which influence human health and well-being” [4]. TR should also include non-scientific sources of knowledge and integrate them throughout the process of research.

In this essay, I present transdisciplinarity as an advanced and specific research tool that can contribute to the new and necessary growth of knowledge in the fields of public health and the environment. I attempt to clarify how researchers may, at least in part, overcome the problem of *incommensurability* (*i.e.*, different disciplines use various languages to explain the same processes or concepts, which leads to incommensurability) between disciplines. I will focus on some aspects of this approach and relate them to the literature of ecosystem services and human social behaviour. I hypothesised that *joint problem defining*, which deals with observations, theories and experiences in a non-hierarchical manner, could result in *joint problem solutions*, as Galliers argued [9]. Joint problem solving would occur across the sciences, technology and society. In the ‘ideal case’, joint problem defining precedes the search for solutions. This process would hypothetically be possible in an open work environment in which transdisciplinarity defines the overarching research structure. I also mention some of the specific problems in complex research. As an introduction, I give an overview of some of the current research issues in health and natural resources related to sustainability and explain why this research requires a new and advanced scientific approach.

## 2. Overview

### 2.1. The Concepts of Health, Mental Health, and Stress

*Concepts of health:* Scientific theories of human health and well being differ widely between disciplines. Biomedical theories of diseases’ aetiology are based on a pathological perspective and, often, on empirical studies of bodily functions. Consequently, health is defined as the state in which bodily mechanisms function correctly according to their biological adaptations. The dysfunction of any organ is defined a state of disease, which should be cured by a biomedical intervention.

However, other theories have emerged in psychology. They are often based partly on hypothetical deduction and partly on controlled observations. The psychological view of disorder usually considers the individual as a complex and dynamic whole, where the interactions between an individual’s personal history and contemporary life may be dysfunctional [10].

In the social sciences, theories of health are frequently based on qualitative or process-oriented research that explores complex relations and tries to put them into models or frameworks. According to these models, ill health is an expression of an imbalance between several interacting systems, and this imbalance may occur on the individual, organisational or social macro level [11].

These perspectives of health and disorder are all, more or less, relevant depending on the context. However, they must be refined and, above all, become more inclusive of the others.

*Mental health and stress:* The global burden of mental health disorders is high and continues to increase [12]. Mental disorders are under-recognised, under-treated and under-prioritised worldwide [13]. According to a report from the World Health Organization (WHO) [14], the worldwide number of deaths by suicide overtook the number of deaths by violence and war in 2002. WHO has claimed that, to reduce the burden of mental disorders, there must be greater attention given to *prevention and promotion* in mental health at the level of policy formulation, legislation, decision-making, resource allocation, and the overall healthcare system.

Many mental diseases are related to stress. The consequences of modern society’s rapid pace of life for human beings are partly unknown, but they include an overload of uncontrollable stress factors. Different organs of the body react in varied ways to stress, and, if these reactions are sustained for a prolonged period of time without the possibility of recovery, they become dysfunctional and risk causing harm to, for example, the cardiovascular and neuro-hormonal systems [15]. One severe symptom of mental distress is impaired cognitive functioning (e.g., communication, concentration, relationships, and empathy) [16], which is especially problematic in light of the difficulties in finding efficient interventions or rehabilitation for these mental states. This difficulty and the lack of adequate health resources, despite the fact that WHO has consistently argued for increased investment in resources for mental health [17,18], are partly reflected by the high rates of long-term sick leaves in Western societies [19]. There is a fundamental mismatch between limited resources, the growing problem of mental diseases, and the growing demand for healthcare. This imbalance mirrors the discrepancy between human biology and our contemporary way of life. Diverse strategies of directing or redirecting health resources have failed, both in scientific and political communities. One reason for this failure may be unsatisfactory theories of what constitutes health and the knowledge we need to maintain it.

### 2.2. Natural Resource Management

Due to the unsustainable exploitation of natural resources for human needs, environmental deterioration and climate change are no longer mere threats, but rather a harsh reality. Many of today’s environmental changes, which were caused by human behaviour, constitute a threat to human health, although the extent of this threat is under debate. In 2000, approximately 5,500,000 disability-adjusted life-years (from malaria, malnutrition, diarrheal disease, heat waves, and floods) were caused by climate change [20].

The imbalance between finite resources and their exploitation has resulted in severe damage to natural landscapes and the loss of biodiversity. Decreased biodiversity is also a potential threat to human health. The possible consequences of decreased biodiversity and destroyed landscapes are the spread of human diseases, loss of medical models, threats to food production and water quality, diminished supplies of raw materials for drug discovery [21], and—not least—restricted areas for recreational use, stress relief and cognitive development. This last issue is important in light of the growing number of persons that suffer from mental diseases. The health implications of biodiversity loss are well summarised in the International Encyclopaedia of Public Health [22].

A proactive environmental and public health response has recently been proposed [23] by building an integrated network for environmental and epidemiological data. This approach is, without doubt, a step in the right direction, but current attempts only focus on Europe. This concept needs to be expanded and applied across borders, especially in light of the unequal distribution of climate change and its consequences.

From a scientific perspective, it is also important to realise that we require not only ecological knowledge but also input from public debate, media and other expressions of society’s view to understand our nature and our behaviour toward environment. This information should be incorporated into the research process.

Researchers have suggested an interesting approach to research in the complex field of diseases related to ecological change that includes, for example, epidemiologic causal criteria, strong inference and triangulation [24]. These techniques also stress the need for cooperative investigations for the sake of healthy populations and ecosystems.

## 3. Ecosystem Services

All human beings are part of ecosystems, and the health and well being of human populations depend on the services these ecosystems provide.

The concept of *Ecosystem Services*, a construct coined by Erlich and Erlich [25], tries to capture the processes by which the environment produces resources that we often take for granted (e.g., clean water, timber, habitats for fish, and the pollination of native and agricultural plants). Whether we are in a city or rural area, ecosystems provide goods and services that are both familiar and fundamental to us.

Unfortunately, the environment’s natural capital and ecosystem services are often underestimated, if they are considered at all. This case has been true for governments, businesses and the public as a whole [26,27]. The importance of ecosystem services is often recognised only after the services have been lost, for one reason or another [28]. However, in the past decade, a movement to value and protect ecosystem services has started [29]. The Millennium Ecosystem Assessment (MA) is one example of this movement; in its vision of the world, natural systems would be appreciated and valued as vital assets because they play a central role in supporting human well being. It has been argued that when people and institutions realise the value of nature for human well-being, they will be motivated to embrace ecological behaviour and increase investments in conservation [29,30]. However, as with any attempt to reframe ecologic concerns in economic terms, we must be aware of potential problems. In some cases, increased investments for conservation are not an appropriate approach, and inadvertent negative consequences may occur [31]. Sometimes, economic profit is neither an accurate nor adequate measure, and it is hoped that a genuine view of ecosystem services would not be limited to the ecosystem’s economic functions [32]. This inadequacy may be one of the reasons why the value of ecosystem services has not yet been clearly defined in the field of ecosystem management [33].

Research has demonstrated the innate positive relation between health, mental state and nature by studying the increased psychological well being that results from contact with natural features and environments [34–36]. Hence, the decreased amount of natural landscapes poses a significant threat to human well being and mental health. In the field of environmental psychology, researchers have studied nature’s importance for our health from the perspective of our evolutionary origins [37,38] and from the perspective of stress, mental fatigue and restoration [39,40]. This research indicates that we have an innate preference for natural environments and that the fascination and non-demanding attention we experience in nature contribute to stress relief.

## 4. Human Social Behaviour

It is important to study human behaviour in this context because it is a commonly held theory that our disconnection from nature and the recent urbanisation of our societies have disrupted the balance between *human biology* and *human behaviour*. We can explore this theory by examining how human behaviour affects our natural surroundings, and vice versa. The results of this research may support the interactive factors between the medical and ecological disciplines.

A possible result of this approach and a potential interactive factor is *human cooperative behaviour*. Cooperation is a fundamental social behaviour, and the cooperative behaviour between humans and between humans and non-humans affects ecosystems and social systems on several levels and in many different ways, pointing towards its importance as an interactive factor between systems.

With the modern neuroimaging techniques [e.g., Positron Emission Tomography (PET), Functional Magnetic Resonance Imaging (fMRI) and Electroencephalography (EEG)] and the mapping of the human genome, it is possible for us to make assumptions about the biological grounding for qualities like cooperation and link them to psychological theories. Below, I briefly review the literature on neuroscience’s contribution to the exploration of cooperative behaviour and discuss how it can be connected to social phenomena.

The concept of *networks* is central to a neurobiological perspective on cooperative behaviour. Neural networks consist of a number of brain areas that carry out psychological functions (e.g., thinking, feeling, and acting) [41].

The executive function *attention* can be seen as the link between psychology and neuroscience. This link describes how voluntary control and subjective experience arise from and regulate our behaviour. The development of attentional functions is partly specified by genes, but is also under influence an individual’s surroundings and culture. The structures of the executive attention network involve specific brain mechanisms, but their function is to influence the operation of other brain networks [42]. Therefore, the performance of this network may affect varied functions such as intelligence [43], personality control [44], the regulation of thoughts and emotions, and conflict resolution [45]—all important aspects of human behaviour. Thus, neural networks, which determine human behaviour, are under the interactive influence of genetic, social and cultural factors and have defined neuroanatomical correlates.

Furthermore, prior social and moral perceptions can influence certain brain structures and networks (e.g., the amygdala and the network of the ventromedial prefrontal cortex, striatum and dopaminergic midbrain) that are involved in *reward* processing and feedback [46]. Biological reward processes can motivate various social behaviours. The reward system consists of components from the mesolimbic dopamine system, including the striatum and the orbitofrontal cortex, and is activated in many different situations. It is also responsible for drug addiction and other addictive behaviours [47].

The neurohormonal basis for cooperative behaviour is also correlated to the brain’s reward system. In short, hormonal feedback loops and systems enable humans to feel pleasure in response to a certain stimulus. When a person achieves a shared outcome through cooperation with another person, his/her reward system is activated, even more so than it would be if the outcome was not linked to another human being. In addition, the dopamine system (an essential part of the reward system) responds negatively if a subject cooperates but the opponent defects [48].

Other clues about the neurobiological basis for social behaviour are sympathy and empathy. Researchers have found the neural correlates of humans’ feelings of sympathy to identify a biological view of how we affectively experience and relate to other people and how such experiences automatically activate an individual’s representation of pro-social behaviour. Furthermore, the neural processes underlying empathy can be modulated by perceptions of other people’s social behaviour; for example, empathy-related brain areas are activated for fair persons but not for persons who are perceived as unfair [49]. Cooperation is usually considered a fair and ethical behaviour, and, as mentioned above, the influence of moral perceptions on the brain structures involved in reward processing [46] stresses the intricate connections between social behaviour, norms and neurological structures. Recently, Barraza and Zak presented further evidence of the biological signatures for empathy [50]. They found that elevated levels of oxytocin (a neurohormone associated with attachment behaviours in mammals) in blood were correlated with feelings of empathy and that higher levels of empathy induced more generous behaviour. Oxytocin also has a direct positive influence on humans’ monetary generosity towards strangers [51]. From this perspective, it is worth mentioning that the same neurohormone, oxytocin, is associated with decreased levels of stress.

Culture and social surroundings have the potential to influence brain functions and behaviour; therefore, we can assume that behaviours can be changed. Motivation is a strong influence on behaviour. The brain’s reward system partially motivates particular behaviours at a biological level, which implies that we could motivate cooperative behaviour by drawing on the potential biological rewards.

For a useful transdisciplinary approach, we require information about the interplay between human health, natural landscapes and ecology. Undoubtedly, there are several possible relationships and interactions, but social behaviours like cooperation could be one important and possibly constructive factor in a transdisciplinary framework to deal with questions of public health and the environment.

*“When we appreciate that two or more very different-seeming phenomena can be treated as similar in some way, we tend to feel that we have accomplished something. This feeling has a simple name: understanding”* [ 52].

Lander, 2010

## 5. Transdisciplinarity as a Method

It has been argued that a transdisciplinary approach is the most suitable approach for understanding health problems that are often embedded in complex causal connections [4,6] and this notion has only grown stronger with time. In addition, there is an increasing need for complex interventions to solve complex problems. Nevertheless, the practice of TR still remains rather scarce.

To meet the demand for a more practically applicable TR, I introduce here a slightly different, or complementary, way of regarding transdisciplinarity: *Inference to the Best Understanding*. This approach is parallel to *Inference to the Best Explanation*, as Harman [53] and Lipton [54], for example, expressed it. This way of regarding TD might be a complement to, or application of, other recent suggestions for research integration, like Integration and Implementation Sciences (I2S) or dialogue methods [55]. It introduces the idea of a firm neurobiological basis upon which further discussions, development, and recursive research may take place in the most integrative way possible. This approach must not be understood as either hierarchical or reductive, but as the multi-faceted approach that is necessary for a deep understanding of issues in which human behaviour plays a key role. In this approach, researchers must be aware of some psycho-neurological prerequisites that must be continuously explored, developed, and integrated and that cannot be overlooked in public health and environmental research.

To a certain extent, research in ecology has presented the idea of inferring conclusions from unique case studies and the ways that informal logic may be associated with this method [2]. The argument for a rationality of practice as a valid research method may be relevant in attempts to apply TR at the boundary between public health and the environment.

By combining diverse disciplines, theories and meta-theories, an *Inference to the Best Understanding* should provide the most coherent worldview possible. This perspective requires the gathering of various theories from different disciplines, and, based on all available data, the process of inference would take place. There is a clear dismantling of the traditional research sequence that leads from scientific insight to action, as Wiesmann *et al.*, for example, suggested [56]. Thus, it is important to take into account the recursive nature of TR [56], which obviates the need for a complete consensus on the initial research questions. However, through the process of inference and with continued iterations and considerations, an increasingly coherent worldview that would be adjustable in space and time could be drawn.

When applying this concept, as is the case with Inference to the Best Explanation [54], a researcher should not aim to explain reality or reach the truth through inference. Rather, the goal is the best understanding of a phenomenon and its manifestations in context, which resonates with Lipton’s concept of loveliness [54]. With understanding as the desired outcome, the results of scientific inquiry become more applicable in society by providing an insight that ought to be useful for setting priorities in contexts ranging from health decisions to natural resource management.

To realize the connection between TR and Inference to the Best Understanding, it is important to treat transdisciplinarity as a specific research instrument and nothing else. The method searches for a synthesis of various connections, and this synthesis is expressed in transdisciplinarity by the focus on interacting factors. These factors are the bridges that make understanding possible, and researchers must thoroughly explore these bridges through TR and the continuous process of inference.

The concept of heterarchy, as opposed to hierarchal structuring, could provide the framework for a transdisciplinarity method like Inference to the Best Understanding. In a heterarchy, the relations between elements and theories are unranked or have the potential to be ranked in numerous ways [57,58]. In combination with Inference to the Best Understanding, the incommensurability between theories would not necessarily, from this perspective, provoke a Kuhnian revolution (According to Kuhn the typical developmental pattern of a mature science is the successive transition from one paradigm to another through a process of revolution. Kuhn argued that a scientific revolution is a noncumulative developmental episode in which an older paradigm is replaced in whole or in part by an incompatible new one); transdisciplinarity would merely be a scientific method that is applicable to any discipline or complex research question. Inevitably, however, this research does not aim to provide explanations or mere descriptions, but aims to provide a view of an issue that is accepted and understood by those involved in the exploring or application process. It is implicit in the concept that this view must be continuously scrutinised, evaluated and reviewed. This critical stance would also contribute to the breakdown of hierarchies and introduce a certain amount of humility; the researchers would accept that whatever the outcome will be it is not more than the best possible and that the optimal outcome could alter with changes in time and space. In addition to offering multiple, simultaneous understandings, equivalent to Lipton’s multiple explanations, continued inference over time has the potential to provide new, refined and more complete insights that place the concept of understanding in a contextual framework. However, this contextualism can be saved from the risk of relativism by a researcher’s commitment to a form of scientific realism [59] that is centred around the best understanding of *human behaviour*. In this approach, the notion and exploration and incorporation of studies in neuroscience and social behaviours are critically and ethically importanct. This perspective accepts the world as unruly and undisciplined; though the brain is actually not, the brain is just infinitely complicated.

In this kind of research team it is in everyone’s interest to seek out the best understanding, and therefore all researchers must strive to overcome incommensurability. Researchers do not need to search for theoretical replacements, but they will be motivated in the search for an increasingly shared vocabulary of understandable words and terms. This shared task gives the researchers a common challenge, which becomes very much part of the research process. This concept of transdisciplinarity does not contradict Kuhn’s view of scientists working in a different world after a paradigm shift [60]. It may be that the world and the view of it changes with further understanding, but this change does not require a fundamental revolution because the concept of incommensurability is eradicated from the process. This approach would offer a more transcendent and inclusive development for science and its expressions, in which the path to the best understanding will also involve different actors from society. Another potential consequence is that the acknowledged uncertainty and complexity of the research process will open science up to the possibility that its theories cover many topics; hence, they become more falsifiable and, in accordance with Popper’s theories, provide better knowledge [ 61].

A transdisciplinary research method has several implications for the ways in which work can be done, and among the most important of these is its time-consuming aspect. To achieve new levels of comprehensiveness, researchers must be aware of the efforts, on an individual and conceptual level, that are necessary. It should be compulsory to work with mutual confidence and trust, and these relationships require time for mutual learning, joint activities and tolerant negotiations to reach at least partial consensus. The method also demands iterations throughout the process so that the team can develop feedback relationships. The iterative and unpredictable nature of TR should be balanced to make the creative inspiration that may arise from such a dynamic process possible; at the same time, the team must stay focused on the integrative factors that were established in the definition phase. To develop the transdisciplinary method scientifically, it is of utmost importance to continue the search for interactive factors and to map inferences at all points along the research path [62].

A true attempt at TR requires researchers to put serious efforts into learning each other’s languages and jargon to reach a shared understanding of the research problem. These efforts would optimally create a language of concepts and models that could be used in specialised and societal contexts. To some extent, so-called systems sciences and complexity theories have adopted the open approach and cross-disciplinary humility [6,63–65], but this approach still needs to be extended and developed.

## 6. Potential Difficulties in Complex Research

Hierarchies: A simplified picture of the differences between disciplines states that medical research has traditionally used quantitative methods and that the social sciences have more often used qualitative ones. The established norms for good quality research in medicine, in which the randomised controlled trial scores highest on the quality hierarchy, are based on quantitative research methods that aim for *evidence-based medicine*. This tradition has blocked attempts to combine disciplines for different aims (e.g., policy setting). When one method is judged as better than another, a counter-productive hierarchy is established and works against the collaborative intellectual work that could cover various scientific traditions.

The science war: There are constructed hierarchies and barriers between disciplines, which have been described as the “two cultures” or the “science war” [66,67].

These barriers mirror the arguments of science as objective or subjective and demonstrate how semantic constructions can work against knowledge production. In the discipline of medicine, knowledge is considered as neutral and more or less objective truths that are derived from controlled and empirical research. This argument is part of the positivistic heritage and establishes quantitative, empirical methods as the foundations of true science. However, the failure of positivistic methods to provide solutions for many complex research questions underlines the need to find new methods of constructing knowledge. Thus, the construction of knowledge and its outcomes becomes more important than the construction of arguments for or against any discipline. Another argument against the construction of fundamental differences between the “two cultures” or “two sciences” is derived from neuroscience, where contemporary theories argue that researchers must use data from various disciplines to gain a more complete understanding of social reactions, emotions, and human behaviour because brain function and plasticity are under the continuous influence of an individual’s environment [68–70].

In contrast, normative methods, such as those presented by Habermas [71], acknowledge the interaction between the researcher’s interests and the research that she/he produces and argue that it is a prerequisite for a true understanding of complex phenomena. This perspective admits that any research method is influenced by the traditions within a discipline, by the surrounding culture and by the researcher’s personality [72]. This argument lies beyond the scope of this paper; however, for interesting and critical reflections on the issue, see Critchley [73].

Reductionism: At least in medicine, the reductionist tradition for the explanation of diseases and methods of intervention has shaped the general mindset and medical education, training and research. This stance is probably due not only to an inbuilt reluctance towards innovative ideas or creativity but also to a tradition in which it is ethically required to be well-informed about details and to work in a structured way to provide advanced and predictable healthcare for patients and to minimise the risks of adverse reactions. Although this approach may have been appropriate when surgical and pharmaceutical interventions were first developed, it is not so in the modern context of health and disease. Thus, the medical establishment must realise that traditional ways of thinking are neither relevant nor ethically defensible when approaching the novel burden of disease.

Incommensurability: Incommensurability can be interpreted as the, per definition, impossibility of making two parallel lines meet. We can apply this metaphor to a scientific paradigm; it is clearly impossible to meet another discipline that develops on another parallel line if there are no cross-roads. Cross-disciplinary thinking is an example of an intersection that may permit some cooperation between the lines, but the disciplines will never truly meet in a constructive way to create novel knowledge. This temporary contact between the sciences can hopefully create a specific outcome, but the methodology and epistemology are not affected in the long-run if there are no true attempts to widen the linear perspectives. Without a common language, the possibility of mutually understandable concepts, or active efforts to redirect the parallel lines into new, intersecting patterns, we cannot expect to raise the current state of knowledge to a higher level. The lack of a shared language can complicate the processes of reaching a consensus on a research question or theory or interpreting research results. If researchers cannot clearly express ideas or phenomena in a complex framework, they cannot properly conceptualise the problem or its outcomes. This problem relates to Kuhn’s view of the incommensurability between disciplines [60]. Kuhn’s later view of incommensurability consists of a translation failure between localised clusters of inter-defined terms in different theoretical languages [74]. Hence, the problem seems to relate mostly to semantics. According to Kuhn, a paradigm consists of predetermined theories with a fixed set of terminology. With the paradigm as a scientific basis, a community of researchers can come together with a mutual logic and language [75]. According to Kuhn, two paradigms are, by definition, incommensurable, and theories from different paradigms cannot be related to each other.

The Kuhnian view of incommensurable paradigms is therefore a major obstacle for this case, considering that we have already established that new knowledge and the integration of varied theories, disciplines and institutions are needed to solve the questions of health and environmental care. This approach excludes linear thinking in exclusive paradigms from research development, and other paths must be explored.

Today’s challenges call for methodological innovations and the creation of an understandable language, potentially in a transdisciplinary context. This context has been formulated and discussed before, not least on the Internet (Network for Transdisciplinary Research, www.transdisciplinarity.ch). However, the debate is fairly young, and further development is still needed.

## 7. Conclusions

The general tendency in the philosophy of science seems to be that knowledge construction becomes more complex as we move from description to causal inference and from applications and interventions to policy change [5]. Unfortunately, a know-do gap (or science-policy gap) exists in public health because there is a gap between what we know about the causes of morbidity and the policy actions taken to mitigate those causes [5]. Hopefully, a broader approach to knowledge seeking can narrow this gap to make research more practical and relevant for policy. This approach should also nurture and empower decisions on environmental policies. Due to scientific uncertainty on environmental issues, it has too often been the case that attempts to implement protective environmental policies have reached no decision. Medical research often shapes policy decisions, and a firmer foundation for policy-making might be laid by linking the relatively grounded knowledge (at least as it has been traditionally seen) that health research produces to these large-scale environmental issues.

There is still an emphasis on biomedical research concerned with therapeutic solutions for individuals with particular diseases. This emphasis is partly due to the fact that most medical research is funded by industry [76], which provides financial incentives for research in pharmacological therapeutic solutions. However, research on complex behavioural interventions of a motivational or preventive character is restricted in terms of funding, academic citing and policy impact. Furthermore, the interactions between natural landscapes, environmental change and health are not easily captured in any sponsor program, and hence research and eventually policy influence are limited. The medical establishment is also much shaped by the Cartesian dualistic heritage; therefore, it is considered a severe biological mistake to believe that the body and soul might have any influence on each other. Applying a transdisciplinary method to these questions is one step towards empowering public health and environmental research with the credibility that is necessary for it to have an impact on policy.

The focus on human social behaviour is interesting from a philosophical point of view because the concept touches on the link between science, society and policies. There has almost been a constant conflict within and between these three arenas about decisions, authority and the proper grounding for decision-making. Policy can be seen as an effort to guide human behaviour [77]. Despite the call for policy grounded in science, policy has failed to change people’s routine behaviours, especially when it comes to health promotion behaviour and natural resource management. Is it possible that these failures occurred because the knowledge on which the policies were based was not relevant? Has this scientific approach not be the ideal approach to provide a consistent worldview?

Exploring the social behaviour of cooperation with a transdisciplinary method may be a step towards integrating knowledge for a relevant and coherent worldview that aims to create policies for sustainable health and environmental care. It should be noted that cooperative behaviour is also one of the most important qualities for a TR project’s success, and, from a policy perspective, a transdisciplinary approach offers the possibility of making the best possible decisions, despite this world’s inevitable uncertainty.
